# Targeted neonatal echocardiography and lung ultrasound in preterm infants with chronic lung disease with and without pulmonary hypertension, screened using a standardized algorithm

**DOI:** 10.3389/fped.2023.1104940

**Published:** 2023-03-23

**Authors:** Janneth Cristina Sánchez-Becerra, Rogelio Guillén-Torres, Rosario Becerra-Becerra, Horacio Márquez-González, Daniel Ibarra-Ríos

**Affiliations:** ^1^ Neonatology Department, National Institute of Health, Hospital Infantil de México Federico Gómez, Mexico City, Mexico; ^2^ Cardiology Department, National Institute of Health, Hospital Infantil de México Federico Gómez, Mexico City, Mexico; ^3^ Clinical Investigation Department, National Institute of Health, Hospital Infantil de México Federico Gómez, Mexico City, Mexico

**Keywords:** lung ultrasonography (LUS), targeted neonatal echocardiography, chronic lung disease, pulmonary hypertension (PH), bronchopulmonar dysplasia, diuretics, furosemide

## Abstract

**Introduction:**

Increased recognition of the development of chronic pulmonary hypertension (cPH) in preterm infants with chronic lung disease (CLD) has prompted enhanced monitoring for the identification of different phenotypes.

**Methods:**

All newborns consulted for oxygen/respiratory support dependency (CLD assessment) from January 2018 to December 2021 were included. TnECHO and LUS screening for cPH-CLD were performed at 36 weeks postmenstrual age. Cases of cPH related to increased pulmonary blood flow (cPH-IPBF) were referred to Pediatric Cardiology. The objective of the study was to identify all cases of cPH (cPH-CLD/IPBF) in the CLD patients screened and to compare outcomes. Following a standardized algorithm, cPH-CLD patients were treated with diuretics; ultrasounds taken before and after treatment were analyzed.

**Results:**

Seventy-two patients with CLD were screened. Twenty-two (30%) had cPH-CLD, and nine (12%) had cPH-IPBF. cPH infants underwent more days of mechanical ventilation, were more likely to have retinopathy of prematurity, and showed increased mortality. The LUS pattern observed in the 72 CLD patients consisted of a thickened pleural line and a B-line interstitial heterogeneous pattern; 29% of patients were found to have lung consolidations. After diuretic therapy, step-down in respiratory support occurred in 59% of neonates with cPH-CLD. A decrease in respiratory rate (RR), right ventricular output (RVO), markers of pulmonary vascular resistance (PVR), and B-line pattern was observed. In tissue Doppler imaging, biventricular diastolic function was found to be modified after diuretics.

**Conclusions:**

CLD infants with cPH showed increased morbidity and mortality. In cPH-CLD patients, a decrease in RR and step-down in respiratory support was observed after diuretic treatment. Follow-up ultrasound showed a decrease in RVO, markers of PVR, and B-lines.

## Introduction

Chronic lung disease (CLD) is the most common respiratory morbidity in surviving preterm newborns. It is a multifactorial disease, and arises as a consequence of factors associated with immaturity of the airway, decreased alveolarization, and decreased lung growth (both airway and pulmonary vasculature) (1).

Reduced vessel density, abnormal vasculature, and vasoreactivity in infants with CLD set the pattern for the development of chronic pulmonary hypertension (cPH). This complication increases with the severity of the CLD and is associated with higher mortality and respiratory and neurodevelopmental morbidity (2). In infants with moderate-to-severe CLD, the incidence of cPH is 30%–50%. This association doubles the risk of mortality (3, 4).

Although cardiac catheterization is the gold standard for diagnosis of pulmonary hypertension, this procedure is invasive, with a risk of complications due to low pulmonary reserve, and is not feasible in developing countries. For this reason, ultrasound has become the most accessible and feasible tool to characterize and follow different phenotypes for physiology-based management (5).

Significant variability exists in clinical practice regarding screening and treatment. Pulmonary edema and right ventricular (RV) congestion play a significant role in the pathophysiology of respiratory symptoms in patients with cPH. Aside from other markers of pulmonary hypertension (which might be variable), a flat interventricular septum in systole with RV dilatation is used to define cPH-CLD. Diuretics have been used to treat alveolar and interstitial edema in neonates, improving RV loading and relieving symptoms (6).

Jain and collaborators at Mount Sinai Hospital in Toronto developed an algorithm in 2014 for screening at-risk infants at 36 weeks postmenstrual age (PMA), where diuretic therapy was the first-line management approach. They used RV dilatation as a therapeutic threshold; this resulted in symptomatic improvement, disease stabilization, and postdischarge outcomes comparable to those of infants with CLD without cPH (6, 7).

## Methods

The location of this study was a Pediatric Tertiary Level Referral NICU in Mexico City with a Targeted neonatal Echocardiography (TnECHO) and Point-of-Care Lung Ultrasound (LUS) program. Our primary objective was to identify all cases of cPH among the CLD patients screened and to compare the in-hospital outcomes of patients with CLD only vs. cPH [including cPH-CLD and increased pulmonary blood flow from ASD/VSD (cPH-IPBF)]. The secondary objectives were to characterize LUS findings in the overall population and to evaluate the results of diuretic therapy (through TnECHO and LUS) in cPH-CLD using a standardized algorithm published in a high-income country.

This retrospective study was approved by the Institutional Research Ethics committee REB HIM 2021-043, and the requirement for informed consent was waived. The study was conducted from January 2018 to December 2021, and included all infants born at <32 weeks of gestational age (GA) with CLD, defined as oxygen/respiratory support dependence at ≥36 weeks PMA in accordance with the National Institute of Child Health and Human Development consensus definition (8). All infants underwent a complete echocardiogram reported by a pediatric cardiologist to rule out structural lesions. Patients were screened and treated according to a previously described standardized algorithm (8). LUS was added as part of the CLD assessment for exploration of the disease characteristics, without influencing treatment decisions.

All scans were performed by a trained staff physician with expertise in TnECHO and LUS. Vital signs from the day before hemodynamic consultation were obtained from the patient chart and averaged. TnECHO and LUS were performed with the dedicated NICU equipment: during 2018 and 2019, this was an Acuson x300™ (Siemens Healthcare, Munich, Germany) with a 9 MHz phased array transducer and a 14 MHz hockey stick probe; during 2020 and 2021, this was a Vivid™ E90 (GE Medical Systems, Milwaukee, WI, USA) with a 12 MHz phased array transducer and an 18 MHz hockey stick probe.

A complete comprehensive echocardiogram was performed in accordance with the 2011 practice guidelines on targeted neonatal echocardiography in the neonatal intensive care unit (9). Tissue Doppler imaging (TDI) was only available for patients evaluated during 2020 and 2021. cPH-CLD was defined as a flat septum in systole (short axis, papillary muscle view) and RV dilatation, diagnosed qualitatively; all cases were managed and followed by the TnECHO team. cPH-IPBF secondary to atrial and ventricular septal defects (ASD, VSD) was managed and followed by the Pediatric Cardiology department and was not included in the before-and-after analysis of diuretic treatment (all >4 mm, with Qp:Qs by echocardiography >1.5:1, RV dilatation and flat septum in systole and diastole).

For LUS, six regions of the anterior and lateral thorax were explored with 6 s clips from medial to lateral longitudinal scans (anterior, inferior, and lateral; right and left). Patients were in a supine position during imaging, so posterior regions were not universally explored (10). A thick pleural line was defined as pleural line >0.5 mm (11).

Pulmonary edema was defined on the basis of qualitative spared areas and B-line count, as follows:

(a) Critical: white lung with coalescent B-lines that obscure A-lines completely; no spared areas.
(b) Severe: 25% spared areas.
(c) Moderate: 50% spared areas.
(d) Mild: 75% spared areas.

Following the recommendations of Jain and collaborators, diuretics were started after cPH-CLD diagnosis. First-line therapy consisted of furosemide 1 mg/kg/dose twice daily if administered intravenously or 2 mg/kg/dose twice daily if administered orally for 5–7 days; this was switched to hydrochlorothiazide and spironolactone if administration of the diuretic induced improvement in respiratory symptoms. Pulmonary vasodilators (sildenafil citrate at 0.5–1.0 mg/kg/dose three times daily) were reserved for cases with worsening clinical symptoms or echocardiography features of cPH despite diuretic therapy (6, 7).

Follow-up ultrasounds were performed 1 week (5–7 days) after diuretic commencement. Respiratory support and step-down were dependent on the attending neonatology team, with a saturation target range of 90%–94%.

Demographic data, management details, and in-hospital outcomes were collected from physical health records. The latest electrolytes taken before treatment and earliest taken after treatment were collected. The radiologic record was reviewed in search of nephrocalcinosis.

Statistical analysis was performed using SPSS Version 25.0 (IBM Corp.). Using normality tests for the quantitative variables, any nonparametrically distributed variables were identified; in these cases, data are expressed in the form of medians and interquartile ranges. Data on electrolytes formed a parametric distribution and are reported in the form of averages and standard deviations. The qualitative variables are expressed as absolute numbers and percentages. Bivariate statistics were performed, using the variable of cPH (present vs. absent) as a comparison group the T test (parametric distribution) or the U Mann Whitney test (non-parametric distribution) were used. Median differences before and after diuretic treatment were examined using the Wilcoxon signed-rank test (quantitative variables). Septal flattening (a qualitative variable) before and after treatment was compared using a linear *χ*^2^ test.

## Results

Seventy-two patients with CLD with a median [interquartile range] gestational age of 29 [27, 31] weeks were screened at 36 [36, 38] weeks PMA. This was a high-risk referral population with deficient prenatal follow-up and with a low rate of prenatal steroids (44.5%). Eighty percent of patients had received surfactant treatment, 68% had early-onset sepsis, and 54% had a healthcare-associated infection. Forty-four percent had a patent ductus arteriosus, with 12% requiring surgical closure. Eighteen percent had severe intraventricular hemorrhage and necrotizing enterocolitis. Ten percent developed retinopathy of prematurity (ROP). Patients with cPH-CLD/cPH-IPBF underwent more days of mechanical ventilation, were more likely to develop ROP, and showed increased mortality before discharge (Table 1). Except for septal flattening in systole with RV dilatation (inclusion criteria), there was no difference in the echocardiographic indices of cPH-CLD/cPH-IPBF patients vs. patients with CLD only.

**Table 1:** Demographics, background, clinical complications of prematurity, and days of respiratory support: comparison of patients with CLD only vs. cPH-CLD/cPH-IPBF.

	CLD only *n* = 41		cPH-CLD/cPH-IPBF *n* = 31		Global *n* = 72	*p*
	*n*/median	%/IQR	*n*/median	%/IQR	*N* [IQR]/(%)	
Maternal age (years)^b^	27	21, 31	26	20, 34	26 [21, 31]	0.9
Maternal hypertension^a^	4	9.8	5	16.1	9 (12.50)	0.3
Maternal diabetes^a^	4	9.8	0	0	4 (5.5)	0.09
Gestational age at birth (weeks)^b^	29	27, 31	29	28, 32	29 [27, 31]	0.2
Intubated at birth^a^	22	53.7	18	58.1	40 (55.50)	0.7
Chest compressions^a^	2	4.9	3	9.7	5 (7)	0.3
5-minute Apgar^b^	8	7, 8	7	6, 8	8 (7, 8)	0.06
Weight (grams)^b^	1,050	880, 1,360	1,010	910, 1,380	1,043 [930, 1,246]	0.9
Female sex^a^	23	56.10	15	50.00	38 (53)	0.6
Cesarean delivery^a^	30	73.20	23	74.20	53 (74.60)	0.4
Antenatal steroids^a^	15	36.60	17	54.80	32 (44.50)	0.1
Surfactant^a^	33	80.50	25	80.60	58 (80.50)	0.9
Early-onset sepsis^a^	27	65.90	22	71.00	49 (68)	0.6
Healthcare-associated infection^a^	20	48.80	19	61.30	39 (54)	0.2
PDA treatment^a^	18	43.90	14	45.20	32 (44.50)	0.9
Surgical PDA closure^a^	7	17.50	2	6.50	9 (12.50)	0.1
Vasopressors^a^	17	42.50	8	25.80	25 (34.70)	0.8
IVH grade 3 and/or PVHI^a^	7	17.10	6	19.40	13 (18)	0.7
NEC ≥ Bell stage 2a^a^	5	12.20	8	25.80	13 (18)	0.1
ROP	1	2.40%	6	19.40%	7 (9.7)	0.001
Postnatal steroids^a^	20	48.70	22	71.00	42 (58.30)	0.09
Mechanical ventilation (days)^b^	12	6, 23	24	14, 31	16 [7, 29]	0.01
nCPAP (days)^b^	15	10, 20	12	8, 21	15 [10, 21]	0.3
High-flow nasal cannula (days)^b^	1	0, 9	4	0, 13	9 [4, 16]	0.1
Low-flow nasal cannula (days)^b^	19	10, 23	13	5, 21	18 [12, 23]	0.03
Oxygen (days)^b^	61	50, 75	61	49, 79	61 [50, 76]	0.7
Hospital stay (days)^b^	62	55, 76	68	55, 88	63 [55, 79]	0.1
Mortality before discharge^a^	1	2.40%	6	19.40%	7 (9.70)	0.001

CLD, chronic lung disease; IPBF, increased pulmonary blood flow; cPH, chronic pulmonary hypertension; PDA, patent ductus arteriosus; IVH, intraventricular hemorrhage; PVHI, periventricular hemorrhagic infarction; NEC, necrotizing enterocolitis; ROP, retinopathy of prematurity; nCPAP, nasal continuous positive airway pressure.

^a^ Fisher’s exact test.

^b^ Mann–Whitney *U* test.

Twenty-two patients (30%) had cPH-CLD and were treated according to the algorithm (6, 7), and nine (12%) were found to have cPH-IPBF (2 VSD, 7 ASD) and referred to Pediatric Cardiology. Of the 32 patients who had previously received PDA treatment at the time of CLD assessment, six had a nonhemodynamically significant PDA.

**Figure 1:**
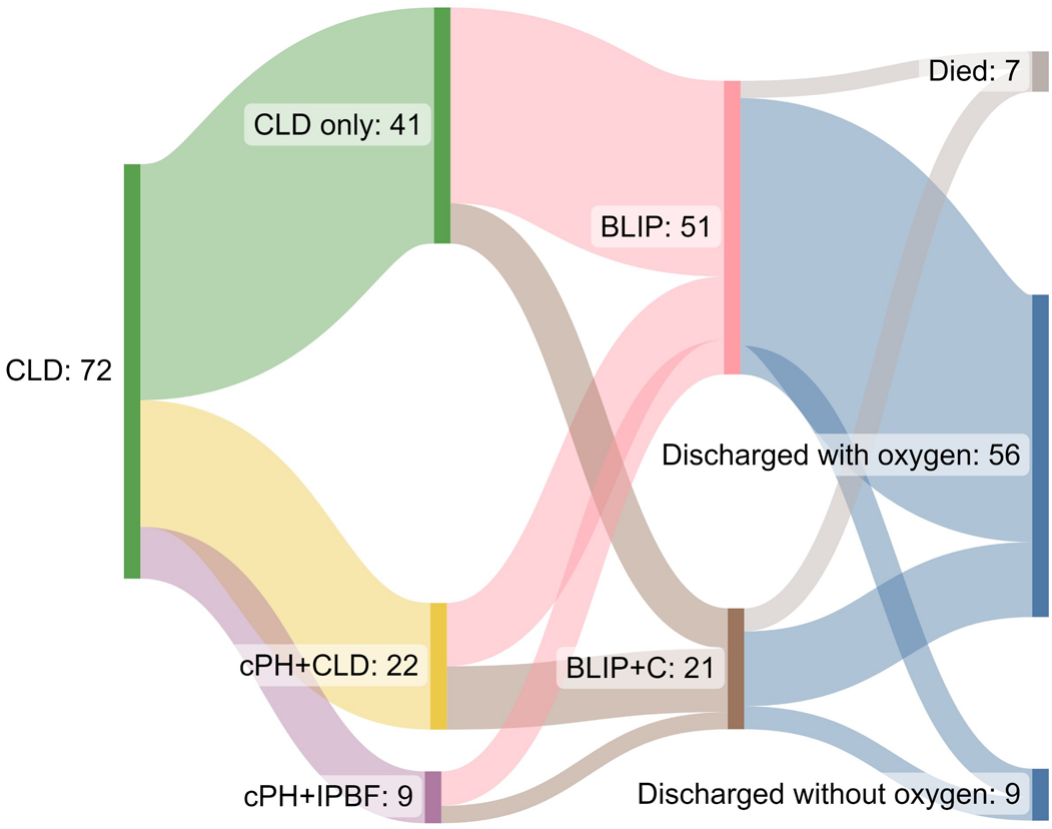
Sankey diagram showing the cardiac and pulmonary phenotypes encountered and discharge information. CLD, chronic lung disease; cPH, chronic pulmonary hypertension; IPBF, increased pulmonary blood flow; BLIP, B-line interstitial pattern; BLIP + C, B-line interstitial pattern with consolidations.

Mortality in our cohort was 9.7%. Fifty-six patients (78%) were discharged with oxygen, and nine (13%) were discharged without oxygen. Figure 1 illustrates the cardiac and pulmonary phenotypes encountered and their outcomes in terms of discharge and mortality.

LUS findings of the 72 CLD patients studied showed a thick pleural line of 0.87 [0.8, 0.9] mm and a B-Line interstitial pneumogenic pattern (BLIP). Twenty-one patients showed lung consolidations (29%), which were reported to the attending team. In twelve patients (16%), a superimposed infectious process was integrated after infectious workup (all with negative bacterial blood cultures; six viral infections were documented: two SARS-CoV-2, two rhinovirus, and two syncytial respiratory virus).

Twenty-two patients with cPH-CLD were treated with diuretics (cPH-IPBF was excluded as follow-up was not carried out by the TnECHO team). At the commencement of treatment, three patients were on mechanical ventilation (14%), seven on nasal CPAP (32%), two on a high-flow nasal cannula (9%), and 10 on a low-flow nasal cannula (45%). In the basal LUS, 59% showed moderate edema (subjectively, 50% spared areas) and 32% showed severe edema (25% spared areas). Table 2 presents the clinical and echocardiographic parameters before and after diuretic therapy, showing a decrease in respiratory rate (RR), RVO, and pulmonary vascular resistance (PVR), demonstrated by pulmonary artery acceleration time (PAAT) alone (which inversely correlates with pulmonary artery pressure) or by PAAT combined with right ventricular ejection time (RVET), referred to as the pulmonary vascular resistance index (PVRi = PAAT/RVET). In 12 of the 22 patients for whom TDI was available, diastolic function was also modified, with an increase in *e*′ velocity from the left and right ventricular free wall. The right ventricular end-diastolic diameter decreased, although the difference did not reach statistical significance. The incidence of flat septum in systole improved from being universal to an incidence of 32% (linear *χ*^2^ test: *p* < 0.001). Disease progression treated with sildenafil occurred in one case. No electrolyte imbalance was found in the patient charts, and there were no reports of nephrocalcinosis in their radiological archives.

**Table 2:** Clinical and echocardiographic parameters before and after diuretic therapy.

*N* = 22 (TDI *n* = 12)	Before		After		*p*
	Median	IQR/SD	Median	IQR/SD	
Clinical^a^					
SAP (mmHg)	66	61, 72	74	67, 83	0.6
DAP (mmHg)	37	34, 42	45	37, 48	0.9
HR (bpm)	158	140, 168	154	143, 163	0.2
RR (rpm)	68	60, 88	62	60, 68	0.01
FiO_2_	30	30, 50	22	22, 28	0.3
Echocardiographic^a^					
**Right ventricle**
RVO (mL/kg/min)	272	228, 379	259	221, 283	0.005
TAPSE (mm)	8.3	7.6, 8.8	9.3	8.54, 10.54	0.7
RV FAC (%)	50	48, 54	46.6	39, 50	0.5
RVET (ms)	190	182, 198	186	175, 200	0.09
PAAT (ms)	50	45, 75	57	49, 69	0.001
PVRi (PAAT/RVET)	0.28	0.23, 0.39	0.29	0.26, 0.37	0.005
RV *e*′ (mm/s)	76	64, 102	107	79, 157	0.018
RV *a*′ (mm/s)	99	86, 107	104	94, 135	0.01
RV *s*′ (mm/s)	68	56, 73	73	72, 87	0.5
RVEDD (mm)	11.9	10.6, 12.51	10.7	9.25, 13.3	0.7
Left ventricle					
LVO (mL/kg/min)	223	184, 242	210	177, 265	0.1
Simpson’s biplane EF (%)	65	61, 68	63	60, 66	0.8
Mitral E (cm/s)	60	60, 69	69	58, 76	0.11
Mitral A (cm/s)	71	55, 78	75	62, 85	0.54
E/A	0.89	0.83, 0.97	0.9	0.79. 7	0.72
IVRT (ms)	52	46, 60	56	50, 69	0.75
LV *e*′ (mm/s)	62	45, 80	78	72, 99	0.01
LV *a*′ (mm/s)	84	70, 94	97	90, 99	0.04
LV *s*′ (mm/s)	57	47, 64	65	56, 72	0.3
Laboratory^b^					
Na (mmol/L)	137.5	4.61	139.65	2.52	0.1
K (mmol/L)	4.79	0.94	4.68	0.53	0.2
Cl (mmol/L)	103.35	6.81	103.29	3.6	0.3

TDI, tissue Doppler imaging; SAP, systolic artery pressure; DAP, diastolic artery pressure; mmHg, millimeters of mercury; HR, heart rate; bpm, beats per minute; RR, respiratory rate; rpm, respirations per minute; FiO_2_, fractional inspired oxygen; RVO, right ventricular output; TAPSE, tricuspid annular systolic excursion; mm, millimeters; RV FAC, right ventricular fractional area change; RVET, right ventricular ejection time; PAAT, pulmonary artery acceleration time; ms, milliseconds; PVRi, pulmonary vascular resistance index; LVO, left ventricular output; EF, ejection fraction; RVDD, right ventricular end-diastolic diameter; mmol/L, millimoles per liter.

^a^ Wilcoxon signed rank test.

^b^ T test.

**Figure 2:**
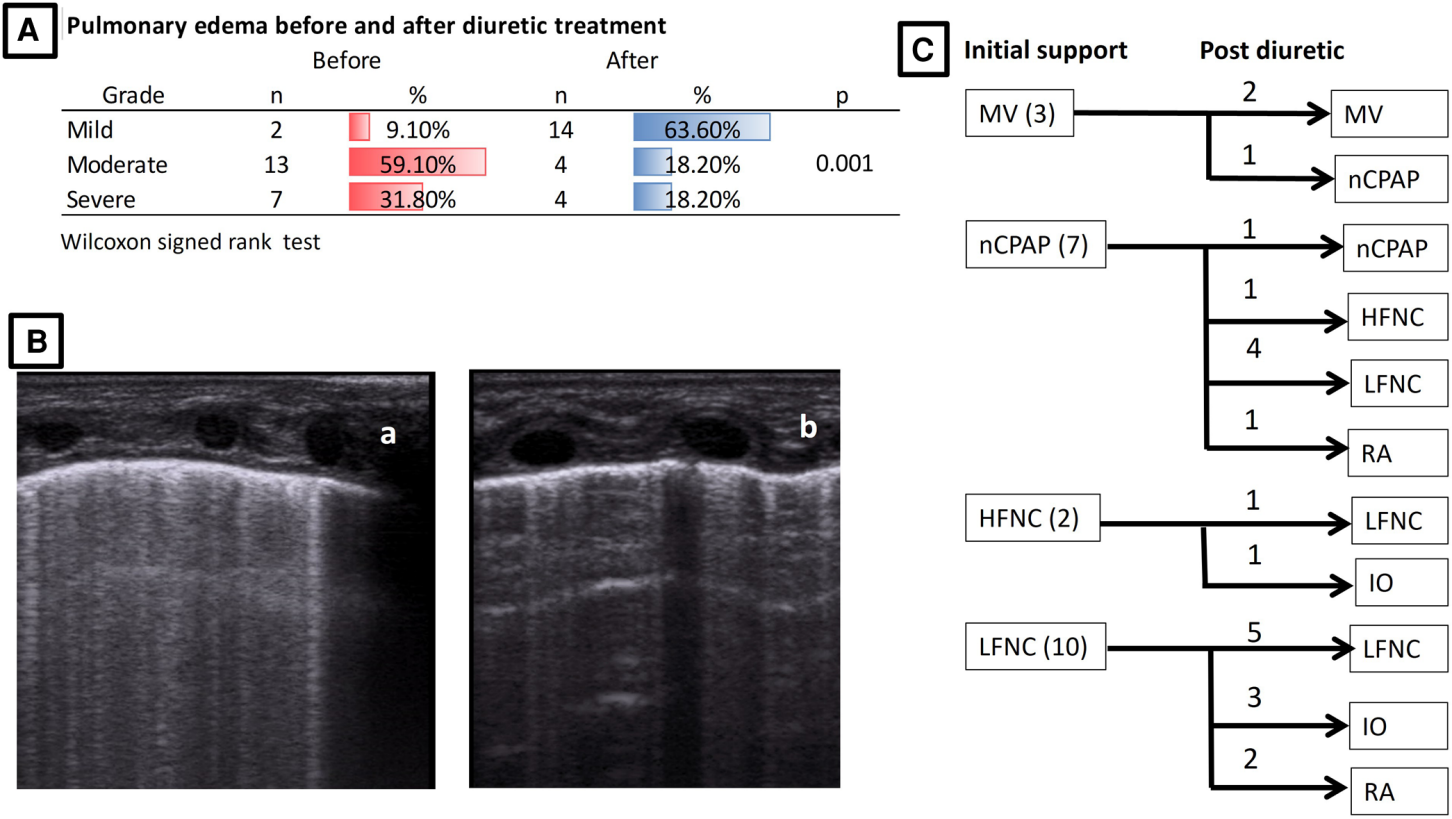
Improvement in pulmonary edema and step-down in respiratory support after diuretic treatment. (**A**) Pulmonary edema before and after treatment. (**B**) LUS example of severe edema (a) that improved to mild edema (b) after diuretic treatment. (**C**) Initial support and step-down after diuretic treatment. MV, mechanical ventilation; nCPAP, nasal continuous positive airway pressure; HFNC, high-flow nasal cannula; LFNC, low-flow nasal cannula; RA, room air; IO, intermittent oxygen.

After diuretic therapy, a step-down in respiratory support occurred in 59% of CLD-cPH neonates. Two patients remained on mechanical ventilation (9%), two on nasal CPAP (9%), 10 on a low-flow nasal cannula (45%), one on a high-flow nasal cannula (5%), and seven on room air/intermittent oxygen (32%). Subjective BLIP count improved after diuretic therapy, increasing the proportion of mild cases from 9% to 64%; the proportion of moderate cases dropped from 59% to 18%, and the proportion of severe cases dropped from 32% to 18%. Figure 2 illustrates this.

## Discussion

With the use of a standardized algorithm, 72 infants were screened as part of our study, identifying 43% with cPH (30.5% cPH-CLD, 12.5% cPH-IPBF); this group showed increased morbidity with more days of mechanical ventilation, a higher incidence of ROP, and increased mortality before discharge. This is concordant with the literature, which shows that around 20% of patients with CLD have cPH; this probability increases with moderate-to-severe CLD (30%–50%), and the condition increases the risk of mortality (4, 12). Nevertheless, there is significant variability in clinical practice regarding screening and treatment (13). Mourani and collaborators showed that early pulmonary vascular disease (based on echocardiograms at 7 days of age) with septal flattening and ventricular dilatation is associated with the development of cPH-CLD, and this can be used to identify high-risk patients (14). Recently, in an ongoing international prospective multicenter observational diagnostic accuracy study, different relevant quantitative echocardiographic indices for early identification of cPH in preterm neonates are being studied (15).

Twelve percent of patients screened were found to have significant shunts (cPH-IPBF). These patients were also treated with diuretics, but were followed up by Pediatric Cardiology. In a large study, Kumar and collaborators showed that the presence of an ASD was associated with an increased probability of developing CLD (16). In another study, Choi et al. demonstrated that the presence of a left-to-right ASD was associated with cPH in preterm infants with moderate or severe CLD (17). In this population, shunt elimination has been shown to improve clinical status and growth in infants with early cPH or those requiring respiratory support (18). It is important to consider ASD or VSD shunts in screening programs for timely referral to Pediatric Cardiology, as some of these shunts detected in early echocardiograms might be missed and acquire physiological importance as CLD develops.

Savoia et al. published a report on a cohort of 23 preterm newborns where CLD follow-up took the form of echocardiography performed by neonatologists during intensive care and in an outpatient clinic. Excellent inter-rater agreement with the cardiology department was found (19). Our hospital started a TnECHO program in 2017, in which CLD assessment represents the fourth leading cause of hemodynamic consultation (20). Since echocardiographic screening was stablished in CLD identifying RV dilatation, diuretics have been used as a physiologically suitable therapy to treat alveolar edema and decrease RV afterload (6, 7). Baczynski et al. showed that screening at ≥36 weeks PMA and use of diuretics as a first-line therapy in newborns with RV dilatation helped to stabilize the disease and improve symptoms (7). Similarly, in our study, symptomatic improvement and a decrease in RR, RVO, and PVR were observed; this allowed a 59% rate of step-down in respiratory support. In Baczynski et al. (7) study, CLD-cPH newborns had postdischarge outcomes comparable to those of infants without cPH; in contrast, our population of cPH-CLD patients (including cPH-IPBF) had increased rates of mechanical ventilation, ROP, and mortality.

PAAT, or PAAT indexed to RVET (to account for the potential impact of heart rate variability), is a noninvasive measure of vascular compliance, resistance, and pressure. Levy and collaborators compared (via regression analysis) echocardiography-derived PAAT against right heart catheterization (RHC)-derived systolic and mean pulmonary artery pressure, PVR index, and compliance in a cohort of 75 children. PAAT inversely correlates with RHC-measured pulmonary pressure and directly correlates with pulmonary arterial compliance in children (21). PAAT increases in preterm newborns during the first year, reflecting the physiological postnatal fall in RV afterload. CLD and cPH exert a negative impact on PAAT measures (22). The fact that this index was modified with diuretic treatment, along with clinical improvement, supports the rationale for its use to decrease alveolar and interstitial edema, improving RV loading.

Normal diastolic function in newborns is difficult to define, especially in the cPH-CLD population. Although none of the patients who received diuretics were considered to have diastolic dysfunction, an improvement was seen. Diastolic function might also play a role in CLD pathophysiology, contributing to reduction of lung compliance as a result of pulmonary venous congestion and pulmonary edema. An increase in right and left ventricular wall *e*′ velocity was observed, probably indicative of an enhancement in ventricular relaxation, since this is less sensitive to loading conditions than pulsed Doppler (23). Despite this statistical difference, diastolic assessment is complex and multifactorial, and it is difficult to assume that the difference represented an improvement. Sehgal et al. have shown impairment of diastolic echocardiographic measures in the CLD population, postulating that postcapillary pathology is a contributor to the overall pathophysiology (24). Their group has also shown that impaired systolic and diastolic RV performance correlates with a longer duration of subsequent respiratory support (25).

LUS has been shown to be a useful tool for predicting CLD (26). Once this is established, it is characterized by a thick pleural line, pneumogenic BLIP, and lung consolidations, as shown in our patients (27). As traditional semiquantitative scores count interstitial pattern as one point regardless of the number of B-lines, we subjectively considered the proportion of spared areas in order to be able to compare patients with universal BLIP. Using a semiquantitative score in preterm infants born before 32 weeks of GA, Alonso-Ojembarrena et al. compared 18 infants before and after diuretic treatment (started on day 31–32). They found changes only in patients who could be weaned off respiratory support (28). In the present study, we found a decrease in pulmonary edema after diuretic treatment. Recently, a case–control study using a modified score also showed that diuretic use is associated with decreased pulmonary edema and improved oxygenation in infants with CLD during the first week of treatment (29). Further prospective research needs to be conducted to explore LUS score progression after diuretic treatment as a suitable biomarker of CLD development and progression. Twenty-nine percent of screened patients presented with lung consolidations, among whom a superimposed infectious process was diagnosed in 16% after the attending team carried out infectious workup (six viral infections). The diagnosis of respiratory viral infections becomes difficult in patients with CLD, since many clinical and radiological diagnostic criteria are part of its natural progression. Viral infections are common among hospitalized CLD infants (30). With the onset of the SARS-CoV-2 pandemic, sampling increased in our institution.

An integrated approach to the detection and treatment of cPH is recommended; nevertheless, this requires high-cost investigations (31). In developing countries, ultrasound is the main available tool, so different markers must be studied for early detection, physiological phenotyping, and monitoring of interventions. We found a standardized algorithm useful for the detection and management with diuretics of CLD patients with cPH. It is important to recognize adverse effects profiles and discontinue medications that are not working. In our study, diuretics were used to improve symptoms and achieve disease stability. As a consultant service, active weaning of diuretics was advised. A recent study showed that active weaning of diuretics did not prolong the duration of home oxygen in the establishment of a standardized clinical guideline (32).

Our study has several limitations, as it consisted of retrospective description and we did not have a comparable untreated group. Another limitation is that our service functions as a consultant service, and we depend on the attending team to ask for hemodynamic consultation. This probably leads to some mild cases going unscreened. During the period in which this study was conducted, only one person performed all the ultrasounds. Step-down in respiratory support was decided by the attending team and was not standardized. Regarding LUS, a semiquantitative scale might be useful, probably one considering posterior regions; we considered spared areas in order to qualitatively characterize a universal BLIP, as we began the study in 2018 and we were not universally calculating semiquantitative scale scores. There was a high rate of use of postnatal steroids in the cPH population (71%), mainly in early disease (before the diuretic course in all cases), which can contribute to step-down in respiratory management, and we were unable to control for this variable. A prospective evaluation of diuretic administration in this population is needed. In future studies, patient safety needs to be addressed. Although we did not document any complications of diuretic use, the study was not designed to identify these, and laboratory evaluation depended on the attending team. A strength of the study is that it provides a real-world depiction of a standardized protocol for the characterization of different clinical phenotypes of CLD in a developing country that describes favorable outcomes and serves to highlight the importance of considering a randomized control trial.

## Data Availability

The raw data supporting the conclusions of this article will be made available by the authors without undue reservation.
